# Research Priorities for Pediatric Emergency Care to Address Disparities by Race, Ethnicity, and Language

**DOI:** 10.1001/jamanetworkopen.2023.43791

**Published:** 2023-11-13

**Authors:** Elyse N. Portillo, Chris A. Rees, Emily A. Hartford, Zachary C. Foughty, Michelle L. Pickett, Colleen K. Gutman, Bashar S. Shihabuddin, Eric W. Fleegler, Corrie E. Chumpitazi, Tiffani J. Johnson, David Schnadower, Kathy N. Shaw

**Affiliations:** 1 Division of Pediatric Emergency Medicine, Baylor College of Medicine, Texas Children's Hospital, Houston; 2 Division of Pediatric Emergency Medicine, Emory University School of Medicine, Children’s Healthcare of Atlanta, Atlanta, Georgia; 3 Division of Emergency Medicine, Department of Pediatrics, University of Washington, Seattle; 4 Section of Emergency Medicine, Department of Pediatrics, Medical College of Wisconsin, Milwaukee; 5 Department of Emergency Medicine, University of Florida, Gainesville; 6 Department of Pediatrics, University of Florida, Gainesville; 7 Division of Emergency Medicine, Nationwide Children’s Hospital, The Ohio State University College of Medicine, Columbus; 8 Division of Emergency Medicine, Boston Children’s Hospital, Boston, Massachusetts; 9 Division of Emergency Medicine, Department of Pediatrics, Duke University School of Medicine, Durham, North Carolina; 10 Department of Emergency Medicine, University of California, Davis, Sacramento; 11 Division of Emergency Medicine, Department of Pediatrics, Cincinnati Children’s Hospital Medical Center and University of Cincinnati College of Medicine, Cincinnati, Ohio; 12 Department of Pediatrics, Perelman School of Medicine, University of Pennsylvania, Children’s Hospital of Philadelphia

## Abstract

**Importance:**

Health care disparities are well-documented among children based on race, ethnicity, and language for care. An agenda that outlines research priorities for disparities in pediatric emergency care (PEC) is lacking.

**Objective:**

To investigate research priorities for disparities in PEC among medical personnel, researchers, and health care–affiliated community organizations.

**Design, Setting, and Participants:**

In this survey study, a modified Delphi approach was used to investigate research priorities for disparities in PEC. An initial list of research priorities was developed by a group of experienced PEC investigators in 2021. Partners iteratively assessed the list through 2 rounds of electronic surveys using Likert-type responses in late 2021 and early 2022. Priorities were defined as achieving consensus if they received a score of highest priority or priority by at least 60% of respondents. Asynchronous engagement of participants via online web-conferencing platforms and email correspondence with electronic survey administration was used. Partners were individuals and groups involved in PEC. Participants represented interest groups, research and medical personnel organizations, health care partners, and laypersons with roles in community and family hospital advisory councils. Participants were largely from the US, with input from international PEC research networks.

**Outcome:**

Consensus agenda of research priorities to identify and address health care disparities in PEC.

**Results:**

PEC investigators generated an initial list of 27 potential priorities. Surveys were completed by 38 of 47 partners (80.6%) and 30 of 38 partners (81.1%) in rounds 1 and 2, respectively. Among 30 respondents who completed both rounds, there were 7 family or community partners and 23 medical or research partners, including 4 international PEC research networks. A total of 12 research priorities achieved the predetermined consensus threshold: (1) systematic efforts to reduce disparities; (2) race, ethnicity, and language data collection and reporting; (3) recognizing and mitigating clinician implicit bias; (4) mental health disparities; (5) social determinants of health; (6) language and literacy; (7) acute pain–management disparities; (8) quality of care equity metrics; (9) shared decision-making; (10) patient experience; (11) triage and acuity score assignment; and (12) inclusive research participation.

**Conclusions and Relevance:**

These results suggest a research priority agenda that may be used as a guide for investigators, research networks, organizations, and funding agencies to engage in and support high-priority disparities research topics in PEC.

## Key Points

**Question:**

Which research topics are most important to medical personnel, researchers, and health care–affiliated community organizations for investigators to prioritize to identify and address disparities in pediatric emergency care?

**Findings:**

In this survey study of 47 family or community and medical or research partners and organizations, 12 priorities were rated highest priority or priority to address disparities in pediatric emergency care by at least 60% of respondents and made up the final research agenda.

**Meaning:**

This study identified 12 priorities that may help individuals funding, conducting, participating in, and publishing research to prioritize lines of investigation in pediatric emergency care disparities research.

## Teaser

This survey study investigates research priorities among medical personnel, researchers, and community organizations to address disparities in pediatric emergency care.

## Introduction

Health care disparities are defined as differences in the quality of health care that are independent of patient need and clinical appropriateness. Disparities occur due to factors, including discrimination, biases, stereotyping, and clinical uncertainty, at the level of the health care system (and the legal and regulatory environment in which it operates) and the level of health care personnel.^1^ Disparities in health care access, clinical management, and health-related outcomes have been well-documented among children based on race, ethnicity, and language for care.^2,3,4^ In pediatric emergency care (PEC), children from communities that are marginalized based on race, ethnicity, or language (such as Black and Hispanic or Latino communities) experience myriad inequities in care, including longer wait times,^5,6^ assignment of lower triage acuity,^7,8,9^ inadequate pain treatment,^10,11,12,13^ and fewer diagnostic imaging tests.^14^ Patients with a language for care other than English are at greater risk for missed diagnoses,^15,16^ delayed treatment for pain,^17^ and unexpected return visits.^18^ Determining the root causes of the many racial, ethnic, and language-based disparities in PEC is complex because of layered effects of long-standing structural racism^19,20^ and individual factors, including implicit and explicit bias.^21,22,23,24^ The high-profile murders of Black individuals in the US and pronounced health disparities identified during the COVID-19 pandemic have increased focus on equity and justice, fueling a sense of urgency to address racism as a root cause of health-related disparities.^25,26,27^

Despite increasing interest in addressing health-related disparities among children in the US, a research agenda in PEC that prioritizes overarching aspects in need of urgent investigation is lacking. While many individual studies have identified disparities, it is imperative that this line of investigation extend beyond identification and description of disparities and move toward reducing and eliminating these disparities. A coordinated effort should focus not only on greater understanding of root causes, but also on developing and implementing interventions and optimizing inclusive and antiracist research practices.^28,29,30,31,32^ Furthermore, a coordinated, multidisciplinary, evidence-based research agenda may inform investigators and funders of research questions in critical need of investigation in PEC.

Our objective was to investigate the most urgent concerns facing minoritized children and adolescents with acute injuries and illnesses treated in the emergency care setting to develop a consensus research agenda. This investigation focused on disparities based on race, ethnicity, and language. While there are additional sources of health disparities, such as disability, gender identity, sexuality, and geographic location, this study’s scope was limited to disparities rooted in race, ethnicity, and language because of the importance and pervasiveness of these factors and to create a focused, actionable research agenda. This research agenda was conceived by the Pediatric Emergency Care Applied Research Network (PECARN) Disparities Working Group (DWG) and is informed by survey responses from investigators, health care personnel (including nurses, nurse practitioners, physician assistants, physicians, and emergency medical service personnel), interpreters, child life specialists, parent representatives, and community members.

## Methods

The Baylor College of Medicine Institutional Review Board (IRB) approved this survey study. The IRB granted a waiver of the requirement for written documentation of informed consent, with implied consent by participants who completed surveys. This study follows the American Association for Public Opinion Research (AAPOR) reporting guideline.

### Objective and Study Design

The objective of this study was to investigate research priorities to identify and address disparities in PEC among medical or research and family or community groups. Study authors are members of the PECARN DWG. PECARN is the first federally funded multiinstitutional network for research in pediatric emergency medicine in the US.^33^ The PECARN DWG comprises PEC physicians, research coordinators, and nurses from across the US dedicated to improving health care for all children, with more than 50 members who represent more than 35 institutions and emergency medical services affiliates and identify across a broad spectrum of nationalities and racial and ethnic backgrounds.

We used a modified Delphi approach to investigate research priorities for racial, ethnic, and language-based disparities in PEC. Previous collaboratives have used modified Delphi processes to establish research agendas within PEC.^34,35^ We followed this precedent and convened a panel of expert partners to provide input using sequential rounds of ranking and scoring.^36^ The study occurred from February 2021 to February 2022.

### Participants

To obtain a representative sample of respondents with varying backgrounds but a shared interest and expertise in PEC, we identified key experts via snowball sampling. An intentional effort was made to include a diverse range of experts from interest groups, research and medical personnel organizations, health care partners (eg, interpreters and child life specialists), and laypersons with roles in community and family hospital advisory councils. Expert partner groups were selected by the PECARN DWG in spring 2021 and characterized as medical or research partner groups and family or community partner groups.

To give equal weight to all partner groups and amplify voices less often heard in research prioritization, this study did not correct for partner group size or numbers of individuals represented by each group. We included a broad range of local, regional, and national groups from across the US and sought input from pediatric emergency research networks outside the US to include their unique perspective in different health systems.

### Survey Development and Dissemination

Survey development took place February to March 2021 and included internal generation of disparities-focused research priorities. We collected initial ideas from PECARN DWG members through a shared online platform, where members could see and comment on each other’s contributions, and an online survey for anonymous submission. This was followed by broader discussion during a virtual forum within PECARN.

We used priorities identified through these internal discussions to generate 2 external surveys: a medical or research partner survey using medical terminology and a family or community partner survey, which adapted the language of the medical or research partner survey for a lay audience (eAppendix 1 and 2 in Supplement 1). PECARN DWG members experienced in survey design (E.N.P., M.L.P., T.J.O., and K.N.S.) made initial revisions to modify the medical or research partner survey into accessible language; a nonmedical lay person then reviewed the survey for readability. Survey instruments and data were managed in research electronic data capture (REDcap), a secure, web-based software platform designed to support data capture for research studies.^37,38^

Surveys were disseminated in 2 iterative rounds. In each survey, partners were asked to review, rate, and provide commentary on proposed priorities. For each round, working group members (including all authors) reached out to partners with an introductory message and a link to the appropriate survey. All partners who completed round 1 were asked to participate in round 2. In these surveys, partners were asked to rate proposed priorities (score range, 1-5, with 1 indicating highest priority for research, 3 listed as neutral, and 5 indicating lowest priority for research). A free-text option to submit new priorities for consideration was included in the round 1 survey only; both surveys included a free-text section for general comments. All survey items were required except the free-text response, thereby precluding missing data within responses. We requested that a single response be submitted for each partner contacted, regardless of size or type of group. If the partner group submitted multiple individual responses instead of a single response, we found the mean of quantitative responses to generate a single response per group. Round 1 occurred August to October 2021 and round 2 occurred November 2021 to January 2022. For each round, up to 3 email reminders were sent to encourage participation.

### Data Analysis

Proposed priorities from round 1 were included in the round 2 survey if they received a score of 1 (highest priority) or 2 (priority) from at least 60% of respondents when examined all together and when broken down by group type (medical or research partners vs family or community partners). The 60% threshold was chosen based on similar studies in literature that used cutoffs between 50% and 80%.^34,39^ Additional priorities suggested during round 1 as free-text proposals were reviewed by the first and senior authors (E.N.P. and K.N.S.). Proposals that were out of the predetermined scope of this research agenda were removed; proposals with a similar theme or focus as existing priorities were combined with those priorities for clarity; and proposals within the scope of the research agenda that had not yet been considered were added as new potential priorities in round 2. Free-text comment responses in both surveys were reviewed for content.

The final agenda included priorities that received a score of 1 (highest priority) or 2 (priority) from at least 60% of respondents. Family or community partners represented a smaller proportion of overall respondents, and thus the 60% threshold was applied separately to ensure adequate representation of viewpoints of these partners. We performed a subanalysis of US-based and non–US-based partners due to feedback that disparities and cultural perceptions of race and ethnicity differ greatly in the US vs other countries. Data were collected in REDCap and then exported to Excel version 2309 (Microsoft Corp) for analysis.

## Results

The workgroup produced a list of 60 partners who were contacted for round 1; among these, 47 partners agreed to participate and received the round 1 survey. Of these, 38 partners (80.6%) scored the proposed priorities, including 29 medical or research partners and 9 family or community partners. Responding partners ranged from local experts to international groups and were geographically diverse (Table 1).

**Table 1.** Partner Respondents

Partner	Location or scope
**Family or community**	
Child Life-Children’s Health Plano	Plano, TX
Language Access Services Children’s Health of Dallas, TX	Dallas, TX
Cincinnati Children’s Research Participant Advisory Council	Cincinnati, OH
Cincinnati Children’s/University of Cincinnati-West End Community Research Advisory Board	Cincinnati, OH
EMSC Family Advisory Network	National, US based
Pediatric Emergency Medicine Interpreter Services, Seattle Children’s Hospital	Seattle, WA
PRIME Node Community Advisory Board, PECARN	Regional: Sacramento, CA, based
**Medical or research**	
National Association of Pediatric Nurse Practitioners	National, US based
Emergency Nurses Association	National, US based
PECARN Research Coordinator Advisory Committee	National, US based
Pediatric Emergency Medicine Collaborative Research Committee	National, US based
National Association of EMS Physicians–Diversity, Equity, Inclusion Committee	National, US based
EMSC Innovation and Improvement Center	National, US based
Society for Pediatric Research	National, US based
Society for Academic Emergency Medicine	National, US based
AAP Committee on Pediatric Emergency Medicine	National, US based
AAP Section on Emergency Medicine	National, US based
Pediatricians Against Racism and Trauma	National, US based
CHAMP E-RNC Buffalo Field Advisory Committee	Buffalo, NY
CHAMP E-RNC Houston Field Advisory Committee	Houston, TX
CHAMP E-RNC Milwaukee Field Advisory Committee	Milwaukee, WI
Hospitals of the Midwest Emergency Research Node investigators	Cincinnati, OH; Milwaukee, WI; St. Louis, MO
Pediatric Emergency Medicine Northeast, West, and South investigators	Denver, CO; Houston, TX; New York, NY
PRIME investigators	Philadelphia, PA; Sacramento, CA; Salt Lake City, UT
San Francisco-Oakland, Providence, Atlanta Research Collaborative investigators	Atlanta, GA; Providence, RI; San Francisco-Oakland, CA
West/Southwest Pediatric Emergency Medicine Research investigators	Dallas, TX; Los Angeles, CA; Seattle, WA
Paediatric Emergency Research in the United Kingdom and Ireland	United Kingdom; Ireland
Research in European Paediatric Emergency Medicine	Europe based
Red de Investigación y Desarrollo de la Emergencia Pediátrica Latinoamérica	South America based
Red de Investigación de la Sociedad Española de Urgencias de Pediatría/Spanish Pediatric Emergency Research Group	National, Spain based

Abbreviations: AAP, American Academy of Pediatrics; CHAMP E-RNC, Charlotte, Houston, Milwaukee Prehospital EMS Research Node; EMS, Emergency Medical Services; EMSC, Emergency Medical Services for Children; PECARN, Pediatric Emergency Care Applied Research Network; PRIME, Pediatric Research in Injuries and Medical Emergencies.

The round 1 survey contained 27 initial priorities. Of these, 18 priorities (66.7%) met inclusion criteria for round 2 (eFigure in Supplement 1). Upon review by the workgroup, 3 priorities that met inclusion criteria and dealt with collection and reporting of race, ethnicity, and language data were combined into 1 new priority. Similarly, 3 priorities that met inclusion criteria focused on language and literacy topics were combined into 1 new priority. After combining similar priorities, 14 priorities from round 1 were included in round 2.

There were also 34 new proposed priorities submitted by partners during round 1, with the following outcomes: there were 20 proposed priorities with similar themes as priorities already meeting inclusion criteria for round 2. These were combined and reworded within this group. An example is the expansion of a priority to recognize and address implicit bias in PEC personnel to include language to “implement bias-reduction strategies.” There were 2 proposed priorities that were disease specific. These were not included in round 2 given that similarly themed priorities received low scores in round 1. (For example, in survey 1, the priority “Asthma assessment and management disparities” received low priority scores, so we did not include a similar proposal for an anaphylaxis-focused priority.) The low priority given to disease-specific priorities was noted in free-text responses by multiple participants. For example, a medical or research partner response in survey round 1 stated, “We believe it is of utmost importance to first have reliable disparities data collection and an understanding of the magnitude of the impact of inequity and disparity on patients as a whole before we look at singular disease processes.” There were 8 proposed priorities outside the predetermined scope to prioritize disparities in PEC based on race, ethnicity, or language that were not included. There were 4 proposed priorities focused on 2 new themes. Therefore, we added 2 new priorities to survey 2: (1) address disparities in the patient and family experience in pediatric care received and as part of clinical and research advisory boards and (2) address the intersectionality of race, ethnicity, and language with other social determinants of health, such as poverty, literacy, and health care access.

Thus, the round 2 survey included 16 priorities: 14 from round 1, all of which had been edited for clarity or combined with other priorities based on feedback from round 1, plus 2 newly identified priorities. The round 2 survey was sent to respondents from round 1. Among 38 partners who responded to round 1, 30 partners (81.1%) responded to round 2, including 23 of 29 medical or research partner groups (79.3%) and 7of 9 family or community partner groups (77.8%).

The final agenda included 12 priorities that met preselected criteria (Table 2). Themes of these priorities were (1) systematic efforts to reduce disparities; (2) race, ethnicity, and language data collection and reporting; (3) recognizing and mitigating clinician implicit bias; (4) mental health disparities; (5) social determinants of health; (6) language and literacy; (7) acute pain–management disparities; (8) quality of care equity metrics; (9) shared decision-making; (10) patient experience; (11) triage and acuity score assignment; and (12) inclusive research participation (Table 2).

**Table 2.** Consensus Research Agenda

Priority	Description
Systematic efforts to reduce inequities	Assess impact of care standardization (evidence-based guidelines, pathways, order sets and other electronic medical record–based tools) on inequity and disparities in the pediatric emergency setting Investigate variations in approach to common sentinel presentations (infants who are febrile, sedation, fracture management, suspected abuse, etc.) and use of standardized tools based on patient race, ethnicity, and preferred language
REAL data collection and reporting	Evaluate and develop best practices for accurately capturing REAL data Improve options for patients who identify with racial or ethnic groups not typically represented in current data-collection schemes Evaluate current REAL data reporting practices and consolidate guidance for pediatric-specific REAL data use and reporting
Recognizing and mitigating implicit bias in PEM clinicians	Identify factors contributing to implicit bias in PEM clinicians Implement bias-reduction strategies, and measure the impact of these strategies
Mental health disparities	Investigate inequities in pediatric emergency mental health service access, medication administration, and de-escalation methods Investigate disparities in mental health disposition and treatment Develop, implement, and assess interventions to reduce disparities in pediatric emergency mental health care Investigate how REAL impact grieving, receipt of bad news, and access to social work or other resources
Social determinants of health	Investigate the interplay between REAL and additional social determinants of health (eg, age, disability, sexual orientation, gender identity, insurance, housing, geographic location, and adverse childhood experiences) Investigate the impact of interventions, such as use of health care navigators and screening and providing resources for social determinants of health, on disparities
Language and literacy in pediatric emergency care	Investigate discrepancies between preferred language and health literacy Implement and test interventions to increase interpreter use Evaluate inequity in consent and discharge instruction availability in non-English languages
Acute pain–management disparities	Investigate sources of disparities in pain management Develop, implement, and assess interventions to reduce inequities and disparities in pain management
Equity in ED quality of care metrics	Evaluate inequities in care provision and process measures in the ED, including clinician wait time, clinician interaction time, and discharge and follow-up communication Evaluate disparities in ED visit outcomes, including visit length and return visits Assess understanding of and compliance with discharge instructions
Shared decision-making	Investigate how to best use shared patient decision-making in pediatric emergency care and research to reduce disparities Develop a tool to measure meaningful engagement of patients and families in pediatric emergency care Assess the impact of health literacy, language, and culture on decision-making of parents and guardians regarding plans of care for their children
Patient experience	Investigate disparities in patient and family experience in pediatric emergency care Implement and assess efforts to include diverse patient and family input in clinical and research advisory boards
Triage and acuity score assignment	Investigate inequities in the triage process that may contribute to disparities in scores by REAL Develop, implement, and assess interventions to reduce inequities and disparities in triage
Research participation	Investigate how patient race, ethnicity, and preferred language impact willingness to participate in research Implement and assess strategies to build trust and show trustworthiness among marginalized populations

Abbreviations: ED, emergency department; PEM, pediatric emergency medicine; REAL, race, ethnicity, and language.

To evaluate the global generalizability of these priorities, we performed a subanalysis of 4 non–US-based partners, which revealed overall agreement in priority scoring between non–US-based and US-based partners. The only priority that did not meet preselected criteria for non–US-based partners was inclusive research participation.

Among included themes, some focused on how disparities research is conducted. Partners prioritized improving collection of race, ethnicity, and language data. It was also clear from comments and scoring that what is studied must include interventions and system changes to address disparities. Illustrative comments on this topic included, “I feel we are at a point where we need some of the efforts to go into actually changing inequities. Yes, the research [is not] perfect in DEI [disparities, equity, and inclusion], but we know there are disparities, and at some point we need action. It’s time for action and change,” and, “Would prioritize starting to do interventional work in these areas to move beyond descriptive research.”

## Discussion

In this survey study, we identified 12 priority areas for addressing racial, ethnic, and language-based disparities in PEC using a modified Delphi process that leveraged expertise of academic, clinical, and community experts. To our knowledge, there have been no published efforts to develop an agenda to guide projects aimed at evaluating and addressing disparities for emergency care of children. In 2003, a research agenda to evaluate and eliminate disparities in general emergency medicine was developed at a specially convened consensus conference,^40^ and a 2023 consensus conference produced an emergency medicine research agenda for addressing racism through health care research, largely focused on adult populations.^41^ Our process identified similar themes to this most recent consensus conference, such as measuring patient experience, evaluating the effectiveness of clinical guidelines to eliminate race-based inequities, and improving representation and participation in emergency department–based research; however, we identified additional areas of focus, such as mental health care disparities, that may be of particular importance for the pediatric population.

Our approach was strengthened by incorporation of community and family partners as experts.^36,42^ Given our focus on racial, ethnic, and language preference–based disparities, we intentionally engaged nonmedical personnel partners in care, as well as family or community members, who may have witnessed or experienced disparate care themselves. This approach is explicitly described as appropriate in Delphi processes, with implications relevant to nonmedical experts like laypeople and community members.^36^

Our unique cohort of diverse experts may make our findings more comprehensive and generalizable. Our research agenda includes multiple patient-centered priorities, including shared decision-making and a focus on the patient experience as central to understanding and addressing disparities in PEC. Addressing disparities in health care, specifically PEC, necessitates collaboration among experts to address structural constructs and factors.^43^ Our findings may foster and advance further cooperation to develop and implement targeted interventions aimed at addressing disparities in PEC.

We found 2 high-scoring priorities, “systematic efforts to reduce inequities” and “race, ethnicity, and language data collection and reporting,” that reflect the dichotomy of perspectives toward approaching disparities research more broadly. The focus on systematic efforts to reduce inequities highlights the call to move beyond documenting disparities toward implementing interventions and making change. However, by prioritizing race, ethnicity, and language data collection and reporting, we acknowledged the importance of high-quality, accurate data in fully understanding the extent of disparities and in assessing efforts that promote equity.^44^ Our final consensus agenda may thus provide a framework for PEC investigators to address disparities from multiple perspectives. Finally, while we chose to focus this research agenda on disparities related to race, ethnicity, and language, free-text comments we received suggested that future work may be needed to establish similar research agendas for disparities related to additional social factors, such as age, disability, sexual orientation, and gender identity.

### Limitations

Our study has several limitations, including that we did not consider, include, or receive complete responses from all experts or key partners that could have participated in this process. Racial and ethnic identity was not collected from members of partner groups; therefore, we cannot guarantee that all racial and ethnic group perspectives are reflected in our findings. Additionally, funding agencies chose not to participate to avoid future conflicts of interest. Despite this, we believe that the degree of participation and our outreach efforts and methods, including providing reminders and sufficient time to complete surveys, allowed us to obtain and analyze a diverse and comprehensive body of responses.

Other limitations include that we did not dictate terms or methods by which groups or agencies would ensure that responses reflected the majority opinion of members. We relied on each contact person to determine what was feasible and most appropriate for the individual group.

As in most consensus studies, our study may have had overrepresentation of some voices or opinions. However, we ensured that our surveys were deidentified and that the initial list supplied by experts in the field was complemented by additional priority items suggested by partners. We systematically collected free-text comments and included select representative comments from participants to capture themes and breadth, but comments were not formally qualitatively analyzed.

## Conclusions

In this survey study of a diverse set of medical and community partners, we established a research priority agenda for disparities in PEC to guide future work in the field. This research agenda may guide individual PEC investigators, research networks, funders, and clinical organizations regarding priority topics for future research. Furthermore, this agenda may inform not only what might be prioritized in disparities research, but also how this may be done. Participants indicated that combining research with quality-improvement strategies and ensuring accurate race, ethnicity, and language data collection are both important as we seek to describe, understand, and mitigate disparities in PEC.

## Notes

Supplement 1. **eFigure.** Process for Developing Research Priorities for Pediatric Emergency Care to Address Disparities by Race, Ethnicity, and Language **eAppendix 1.** Survey 1 **eAppendix 2.** Survey 2

Supplement 2.
Data Sharing Statement
