# Supplementary material for: Research Priorities for Pediatric Emergency Care to Address Disparities by Race, Ethnicity, and Language
Source: JAMA Netw Open. 2023 Nov 13;6(11):e2343791. doi: 10.1001/jamanetworkopen.2023.43791 (PMC10644218; doi:10.1001/jamanetworkopen.2023.43791)
Supplement: Supplement 1. — eFigure. Process for Developing Research Priorities for Pediatric Emergency Care to Address Disparities by Race, Ethnicity, and Language eAppendix 1. Survey 1 eAppendix 2. Survey 2 [file jamanetwopen-e2343791-s001.pdf]

Supplemental Online Content

Portillo EN, Rees CA, Hartford EA, et al. Research priorities for pediatric emergency care to address disparities by race, ethnicity, and language. *JAMA Netw Open*. 2023;6(11):e2343791. doi:10.1001/jamanetworkopen.2023.43791

eAppendix 1. Survey 1
eAppendix 2. Survey 2
eFigure 1. Process for Developing Research Priorities for Pediatric Emergency Care to Address Disparities by Race, Ethnicity, and Language

This supplemental material has been provided by the authors to give readers additional information about their work.

© 2023 Portillo EN et al. *JAMA Network Open.*

eAppendix 1: Survey 1

Please note, survey instruments were sent via email with an introductory message and delivered using REDCap. The survey instrument had radio buttons for participants to select their scores for each priority. The text of the body of the survey instrument is included below.

Medical/Research Partner Survey 1: Disparities in Pediatric Emergency Care Research Agenda

We are working to prioritize a research agenda for healthcare inequities and outcome disparities in pediatric emergency care. We are considering research priorities in the domains of: Identifying Disparities, Understanding Sources of Disparities, Developing and Testing Interventions Aimed at Improving Disparities, Research Process and Methods, and Workforce Diversity.
Please complete the survey below. NOTE: You may save and return to the survey if needed.
Thank you!

Please rank each of the proposed items below with a score between 1 (highest priority) and 5 (lowest priority) based on your group's input.

1. Investigate how to best use shared patient decision making in network research to reduce disparities
2. Race, ethnicity, and language (REaL) data collection and core elements: Defining best practices for PEM-essential data elements AND how they're collected
3. Improving data options and collection for patients who identify with minority groups based on their gender, race, ethnicity, etc. (for example, patients whose race/ethnicity is not currently represented in large databases except as "Non-Hispanic-other")
4. Evaluating and developing best practices for reliably capturing Limited English Proficiency (LEP), preferred language, and interpreter use data
5. Recognizing and mitigating implicit bias in PEM providers
6. Triage/acuity assignment inequity by preferred language/race/ethnicity
7. Interventions to increase interpreter use
8. Understanding obstacles to interpreter use
9. Impact of inequity/disparity data visibility (i.e. dashboard) on provider perspectives
10. Asthma assessment and management disparities
11. Sepsis assessment and management disparities
12. Inequity in consent availability in non-English languages
13. Inequity in restraint use by preferred language/race/ethnicity
14. Disparities in sedation consent
15. Disparities in acute pain management
16. Disparities in mental health service access, medication, de-escalation, disposition
17. Disparities in mTBI presentation and follow up
18. Assessing discrepancies in preferred language and health literacy
19. Understanding how race/ethnicity impacts willingness to participate in research
20. Understanding how race/ethnicity of researchers impacts patients' willingness to participate
21. Diversity in research teams
22. Inequity in EMS practices (such as type of dispatch response, decision to transport, restraint use, and protocol adherence)
23. Disparities in prehospital morbidity and mortality
24. Impact of governmental policies on reducing disparities
25. Impact of care standardization (evidence-based guidelines, pathways, order sets) on inequity and disparities

26. Inequities in provision of care in the ED including visit length, provider interaction time, and discharge and follow-up communication by preferred language/race/ethnicity
27. Understanding how race/ethnicity impacts grieving, receipt of bad news, access to social work or other resources

Please use this space to include any comments you have on the priorities above. Please indicate the priority number(s) if referring to a specific priority or priorities.

Please use this section to submit additional priorities for consideration that your group would like us to consider but did not see above.

Family/Community Partner Survey 1: Disparities in Pediatric Emergency Care Research Agenda

A group of pediatric emergency medicine researchers are working to create a list of possible future research priorities (a "research agenda") for healthcare inequities and outcome disparities in pediatric emergency care (see definitions below). We would like your assistance ranking these priorities.
Healthcare inequities: Health inequities are differences in health status or in the distribution of health resources between different population groups, arising from the social conditions in which people are born, grow, live, work and age. Health inequities are unfair and could be reduced by the right mix of government policies. (www.who.int)
Outcome Disparities: Health disparities are preventable differences in the burden of disease, injury, violence, or in opportunities to achieve optimal health experienced by socially disadvantaged racial, ethnic, and other population groups, and communities. (www.cdc.gov)
Please complete the survey below indicating the responses for your group (one response per group, please). If you feel there is an item that you either do not have enough information to score or do not feel that your group has an opinion about, you can leave it blank.
Thank you!

Please rank each of the proposed items below with a score between 1 (highest priority) and 5 (lowest priority) based on your group's input. NOTE - Background information for items #10-17 below: There is research that shows there are disparities and inequities in the following areas. For each area, what is the priority for future research to find the source of these problems and to improve them.

1. Investigate how to best use shared decision making* in research studies to reduce disparities. [*Shared decision making is a process in which clinicians and patients work together to make decisions and select tests, treatments and care plans based on clinical evidence and patient preferences and values (healthit.gov)]
2. Figuring out the best way to collect information about patients' race, ethnicity, and language
3. Improving options to describe patient identity based on their gender, race, ethnicity, etc. (For example, giving more options for patients who typically select "other" for their racial identity.)
4. Improving the way we determine preferred language for patients in research studies, and which patients have a limited understanding of English and/or need interpreters
5. Recognizing and reducing implicit (subconscious or unconscious) bias in Pediatric Emergency Medicine providers
6. Making sure patients in the emergency department are seen based on how sick they are regardless of their race, ethnicity, or preferred language
7. Figuring out ways to increase the use of interpreters for those who need interpreters
8. Understanding obstacles to using interpreters

© 2023 Portillo EN et al. *JAMA Network Open.*

9. Researching how provider behaviors change when they're made aware of inequities or disparities during or after providing care
10. Asthma management
11. Sepsis* management [*Sepsis is a serious condition that can occur when a patient's body is responding to an infection. It can lead to organ disfunction and potentially death]
12. Obtaining consent for patients who do not speak English
13. Using restraints for patients who may harm themselves or others
14. Obtaining consent for sedation* [*Sedation is medication to calm a patient or put them to sleep so that a test or procedure can be done]
15. Acute pain management
16. Mental health services
17. Concussion management
18. Assessing differences between a patient's preferred language and their ability to understand health information and make health decisions
19. Understanding how an individual's race/ethnicity impacts their willingness to participate in research
20. Understanding how a researcher's race/ethnicity impacts patients' willingness to participate in research
21. Diversity in research teams
22. Differences in how first responders (like paramedics) manage patients
23. Differences in sickness and death that occur before a patient reaches the emergency department based on factors like their age, geography (rural vs. urban), race/ethnicity, and preferred language
24. Impact of governmental policies on reducing disparities
25. Impact of standardizing medical care (like following guidelines) on inequity and disparities
26. Differences in how care is provided in the ED (including visit length, time with a provider, and discharge and follow-up instructions) based on patient preferred language/race/ethnicity
27. Understanding how race/ethnicity impacts grieving and receiving bad news, and access to social workers or other resources (like a chaplain)

Please use this space to include any comments you have on the priorities above. Please indicate the priority number(s) if referring to a specific priority or priorities.

Please use this section to submit additional priorities for consideration that your group would like us to consider but did not see above.

© 2023 Portillo EN et al. *JAMA Network Open.*

eAppendix 2: Survey 2

Please note, survey instruments were sent via email with an introductory message and delivered using REDCap. The survey instrument had radio buttons for participants to select their scores for each priority. The text of the body of the survey instrument is included below.

Medical/Research Partner Survey 2: Disparities in Pediatric Emergency Care Research Agenda

We are working to prioritize a research agenda for healthcare inequities and outcome disparities in pediatric emergency care based on race, ethnicity, and preferred language. We are considering research priorities in the domains of: Identifying Disparities, Understanding Sources of Disparities, Developing and Testing Interventions Aimed at Improving Disparities, Research Process and Methods, and Workforce Diversity.
Please complete the survey below by January 21, 2022. NOTE: You may save and return to the survey if needed.
Thank you!

Please assign each of the proposed items below a score between 1 (highest priority) and 5 (lowest priority) based on your group's input (if submitting a group response) or based on your priorities (if submitting an individual response). All the following research priorities apply to the pediatric emergency care setting unless otherwise specified. For the purposes of this project, our focus is on inequities and disparities related to race, ethnicity, and language preference (referred to below as "REaL").

1. Race, Ethnicity and Language (REaL) Data Collection and Reporting: Evaluate and develop best practices for accurately capturing race, ethnicity, and language (REaL) data; Improve options for patients who identify with racial or ethnic groups not typically represented in current data collection schemes; Evaluate current REaL data reporting practices and consolidate guidance for pediatric-specific REaL data use and reporting
2. Recognizing and Mitigating Implicit Bias in PEM Providers: Identify contributing factors to implicit bias in PEM providers; Implement bias-reduction strategies, and measure the impact of these strategies
3. Triage/Acuity Score Assignment: investigate inequities in the triage process that may contribute to disparities in scores by REaL; develop, implement, and assess interventions to reduce inequities/disparities in triage
4. Mental Health: Investigate inequities in pediatric emergency mental health service access, medication administration, and de-escalation methods; investigate disparities in mental health disposition and treatment; develop, implement, and assess interventions to reduce disparities in pediatric emergency mental healthcare; Investigate how REaL impacts grieving, receipt of bad news, access to social work or other resources
5. Systematic Efforts to Reduce Inequities: Assess impact of care standardization (evidence-based guidelines, pathways, order sets and other electronic-medical-record-based tools) on inequity and disparities in the pediatric emergency setting; investigate variations in approach to common sentinel presentations (febrile infants, sedation, fracture management, suspected abuse, etc.) and use of standardized tools based on patient race, ethnicity, and preferred language
6. Disparities in Acute Pain Management: Investigate sources of disparities in pain management; develop, implement, and assess interventions to reduce inequities/disparities in pain management
7. Emergency Department Quality of Care Metrics: Evaluate inequities in care provision in the ED including provider wait time, provider interaction time, and discharge and follow-up

© 2023 Portillo EN et al. *JAMA Network Open.*

communication; Evaluate disparities in ED visit outcomes including visit length and return visits; Assess understanding of and compliance with discharge instructions

8. Pre-hospital and Inter-hospital Practices: Evaluate inequity in EMS practices (such as type of dispatch response, decision to transport, restraint use, and protocol adherence); Evaluate disparities in pre-hospital law enforcement involvement; Evaluate the impact of REaL on inter-facility transfers to specialty hospitals
9. Shared Decision Making: Investigate how to best use shared patient decision making in pediatric emergency care and research to reduce disparities; Develop a tool to measure meaningful engagement of patients and families in pediatric emergency care; Assess the impact of health literacy, language, and culture on decision-making of parents/guardians regarding plans of care for their children
10. Restraint and Security Practices: Evaluate inequity in medical and physical restraint use; Evaluate disparities in use of hospital security and law-enforcement presence for pediatric emergency patients
11. Clinical and Research Team Characteristics: Evaluate how the clinical team's (nurses, advanced practice providers, physicians, etc.) race/ethnicity/language impacts patient care for minoritized populations; Evaluate how research team race/ethnicity/languages spoken impacts minoritized patients' willingness to participate in research; Investigate how leadership diversity, policies, and programs impact diversity of clinical and research teams
12. Language and Literacy in Pediatric Emergency Care: Investigate discrepancies between preferred language and health literacy; Implement and test interventions to increase interpreter use; Evaluate inequity in consent and discharge instruction availability in non-English languages
13. Research Participation: Investigate how patient race/ethnicity/preferred language impacts willingness to participate in research; Implement and assess strategies to build trust and show trustworthiness among marginalized populations
14. Policy Impact: Investigate the impact of governmental (federal, state, and local) policies on reducing health disparities; Investigate the impact of institutional policies on reducing health disparities
15. Patient Experience: Investigate disparities in patient and family experience in pediatric emergency care; Implement and assess efforts to include diverse patient and family input in clinical and research advisory boards
16. Social Determinants of Health (SDoH): Investigate the interplay between REaL and additional social determinants of health (age, disability, sexual orientation and gender identity, insurance, housing, geographic location, adverse childhood experiences, etc.); Investigate the impact of interventions such as use of healthcare navigators and screening and providing resources for SDoH on disparities

Please use this space to include any comments you have on the priorities above. Please indicate the priority number(s) if referring to a specific priority or priorities.

© 2023 Portillo EN et al. *JAMA Network Open.*

Family/Community Partner Survey 2: Disparities in Pediatric Emergency Care Research Agenda

As you may recall from survey 1, a group of pediatric emergency medicine researchers are working to create a list of possible future research priorities (a "research agenda") for healthcare inequities and outcome disparities in pediatric emergency care (see definitions below) based on race, ethnicity, and preferred language. We would like your assistance ranking these priorities.
Healthcare inequities: Health inequities are differences in health status or in the distribution of health resources between different population groups, arising from the social conditions in which people are born, grow, live, work and age. Health inequities are unfair and could be reduced by the right mix of government policies. (www.who.int)
Outcome Disparities: Health disparities are preventable differences in the burden of disease, injury, violence, or in opportunities to achieve optimal health experienced by socially disadvantaged racial, ethnic, and other population groups, and communities. (www.cdc.gov)
The following survey contains items that scored highly on survey 1, with some edits and additions suggested by participants.
Please complete the survey below based on your group's input (if submitting a group response) or based on your priorities (if submitting an individual response) by January 21, 2022.
NOTE: You may save and return to the survey if needed.
Thank you!

Please assign each of the proposed items below a score between 1 (highest priority) and 5 (lowest priority) based on your group's input (if submitting a group response) or based on your priorities (if submitting an individual response). All the following research priorities apply to the pediatric emergency care setting unless otherwise specified. For the purposes of this project, our focus is on inequities and disparities related to race, ethnicity, and language preference (referred to below as "REaL").

1. Race, Ethnicity and Language (REaL) Data Collection and Reporting: Figure out the best way to collect information about patients' race, ethnicity, and language preferences (REaL); Improve options for patients to describe their identity (for example, giving more options for patients who typically select "other" for their racial identity); Evaluate how this information is currently collected and reported and provide guidance for future collection and reporting
2. Recognizing and Reducing Implicit Bias in Pediatric Emergency Medicine (PEM) providers: Figure out how, why, and when PEM providers unconsciously use implicit bias (stereotypes); Design programs that prevent providers from using bias or stereotypes
3. Triage/Acuity Score Assignment: Make sure patients in the pediatric emergency department are seen based on how sick they are regardless of their race, ethnicity, or preferred language
4. Mental Health: Study disparities in pediatric emergency mental health care; design programs to reduce disparities in pediatric emergency mental healthcare; Study differences in grieving and receipt of bad news, and differences in access to social work and other resources
5. Systematic Efforts to Reduce Inequities: Study whether guidelines and national standards reduce disparities in the pediatric emergency setting; Investigate differences in approaches to similar conditions for patients with different races, ethnicities, preferred languages
6. Disparities in Acute Pain Management: Study disparities in pain management; design programs to reduce disparities in pain management
7. Emergency Department Quality of Care Metrics: Study differences in how care is provided in the ED (including wait times, time with a provider, and discharge and follow-up instructions) and outcomes like visit length and return visits; Study patient understanding and ability to follow discharge instructions

© 2023 Portillo EN et al. *JAMA Network Open.*

8. Pre-hospital and Inter-hospital Practices: Evaluate differences in how first responders (like paramedics) manage patients; Evaluate differences in police interactions with patients before they arrive to the ED by REaL; Study disparities in transfers to specialty hospitals
9. Shared Decision Making: Study how PEM providers can work with patients and families when making decisions (also called shared decision making) and how this can affect disparities. Study how language, culture, and ability to understand health information impact how patients and families make decisions. Design a program to help patients and families understand different health decisions
10. Restraint and Security Practices: Study differences in restraint use (both physical restraints and sedating medicines) for patients who may harm themselves or others; Study differences in in use of hospital security and police presence for pediatric emergency patients
11. Clinical and Research Team Characteristics: Study how the clinical team's race/ethnicity/language impacts care for patients from minority groups; Study how research team race/ethnicity/languages spoken impacts patient and family willingness to participate in research; Study how leadership diversity, policies, and programs impact diversity of medical and research teams
12. Language and Literacy in Pediatric Emergency Care: Study differences between a patient's preferred language and their ability to understand health information and make health decisions; Try to increase interpreter use for those who need them; Study differences in consent form and discharge instruction availability in non-English languages
13. Research Participation: Study how patient race/ethnicity/preferred language affects the decision to participate in research; Design programs to build trust and show trustworthiness among populations that have been excluded from research or are hesitant to participate
14. Policy Impact: Study the impact of governmental (federal, state, and local) policies on reducing health; Study the impact of institutional policies on reducing health disparities
15. Patient Experience: Study disparities in patient and family experience in pediatric emergency care; Assess efforts to include diverse patient and family input in clinical and research advisory boards
16. Social Determinants of Health (SDoH): Study the interaction between REaL and additional SDoH; Study programs that provide resources for SDoH; [Social determinants of health (SDoH) are the conditions in the environments where people are born, live, learn, work, play, worship, and age that affect a wide range of health, functioning, and quality-of-life outcomes and risks. (https://health.gov/healthypeopl e/objectives-and-data/social-determinants-health)]

Please use this space to include any comments you have on the priorities above. Please indicate the priority number(s) if referring to a specific priority or priorities.

© 2023 Portillo EN et al. *JAMA Network Open.*

eFigure: Process for Developing the Research Priorities for Pediatric Emergency Care to Address Disparities by Race, Ethnicity, and Language

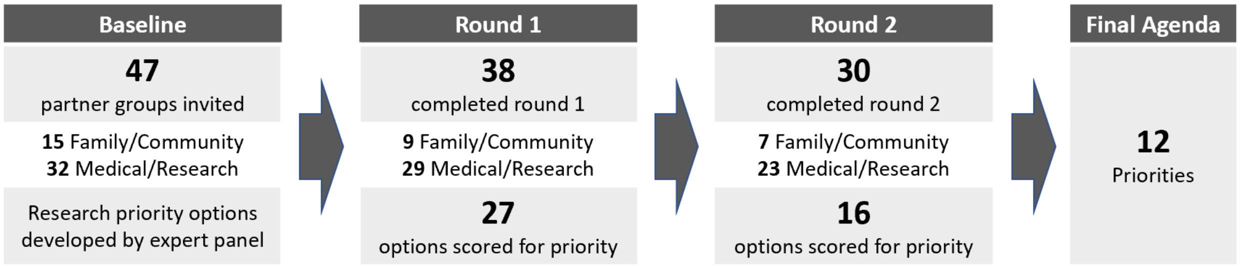

© 2023 Portillo EN et al. *JAMA Network Open.*
