# Supplementary material for: Research Priorities for Pediatric Emergency Care to Address Disparities by Race, Ethnicity, and Language
Source: JAMA Netw Open. 2023 Nov 13;6(11):e2343791. doi: 10.1001/jamanetworkopen.2023.43791 (PMC10644218; doi:10.1001/jamanetworkopen.2023.43791)
Supplement: Supplement 2. — Data Sharing Statement [file jamanetwopen-e2343791-s002.pdf]

Data Sharing Statement

Portillo. Research Priorities for Pediatric Emergency Care to Address Disparities by Race, Ethnicity, and Language. *JAMA Netw Open*. Published online November 15, 2023. doi:10.1001/jamanetworkopen.2023.43791

Data
Data available: No
